# Red-Shifted Excitation and Two-Photon Pumping of Biointegrated GaInP/AlGaInP Quantum Well Microlasers

**DOI:** 10.1021/acsphotonics.1c01807

**Published:** 2022-02-16

**Authors:** Vera M. Titze, Soraya Caixeiro, Andrea Di Falco, Marcel Schubert, Malte C. Gather

**Affiliations:** † SUPA, School of Physics and Astronomy, University of St Andrews, North Haugh, St Andrews KY16 9SS, United Kingdom; ‡ Humboldt Centre for Nano- and Biophotonics, Institute of Physical Chemistry, University of Cologne, Greinstr. 4-6, D-50939 Cologne, Germany

**Keywords:** microlasers, quantum wells, III−V semiconductors, cell tracking, two-photon excitation, biolaser

## Abstract

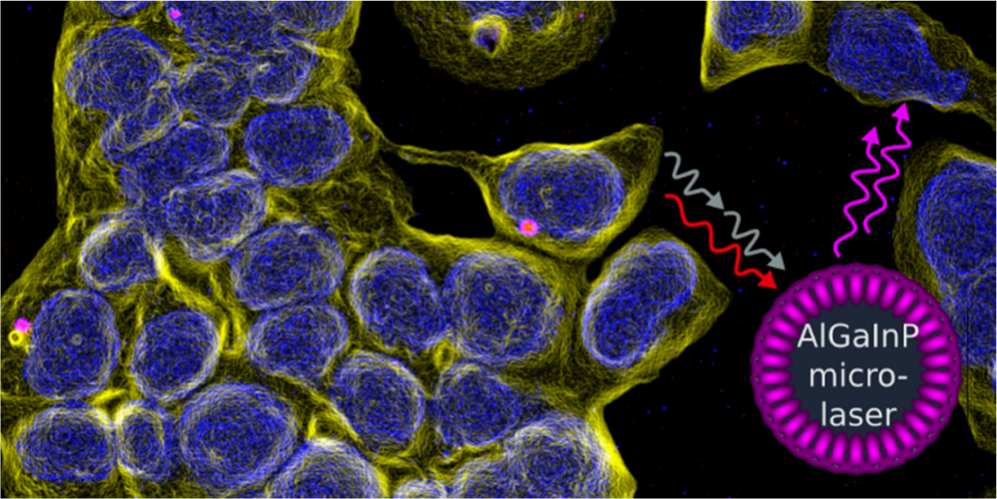

Biointegrated intracellular microlasers have emerged as an attractive and versatile tool in biophotonics. Different inorganic semiconductor materials have been used for the fabrication of such biocompatible microlasers but often operate at visible wavelengths ill-suited for imaging through tissue. Here, we report on whispering gallery mode microdisk lasers made from a range of GaInP/AlGaInP multi-quantum well structures with compositions tailored to red-shifted excitation and emission. The selected semiconductor alloys show minimal toxicity and allow the fabrication of lasers with stable single-mode emission in the NIR (675–720 nm) and sub-pJ thresholds. The microlasers operate in the first therapeutic window under direct excitation by a conventional diode laser and can also be pumped in the second therapeutic window using two-photon excitation at pulse energies compatible with standard multiphoton microscopy. Stable performance is observed under cell culturing conditions for 5 days without any device encapsulation. With their bio-optimized spectral characteristics, low lasing threshold, and compatibility with two-photon pumping, AlGaInP-based microlasers are ideally suited for novel cell tagging and *in vivo* sensing applications.

Over the past decade, biointegrated microlasers have emerged as an attractive alternative to the fluorescent labeling of cells.^1−6^ Compared to techniques based on quantum dots or fluorescent probes, lasers have significantly advanced our ability to track large quantities of cells, principally as a result of their narrow linewidth emission and unique emission spectrum, which can be used as an optical barcode. In addition, the sensitivity of these microlasers to minute changes in the refractive index of the immediate environment makes them excellent micro- and nanoscale biosensors.^7−10^ So far, the field is dominated by organic materials—typically polymers, oils, or liquid crystals loaded with fluorescent dyes—which prevents miniaturization of the optical cavity to dimensions below a characteristic size of approximately 10 μm due to the weak refractive index contrast between the laser (*n*_cavity_ ∼ 1.6–1.8 for many organic materials) and the cell (*n*_cell_ ∼ 1.38).^11,12^ Such optical cavities are comparable in size with the nucleus of eukaryotic cells and the presence of these microlasers might consequently affect cellular functions. The introduction of inorganic semiconductor materials has recently enabled the fabrication of sub-micrometer-sized intracellular laser probes as their higher refractive index (*n* ∼ 3.3–3.6) allows for smaller cavity sizes.^4,6,13^ These lasers bear further advantages, such as excellent photostability and low pump thresholds. Starting from wafers of the respective semiconductor, disk-shaped whispering gallery mode (WGM) microresonators of well-controlled size can be readily produced using photo- or electron-lithography and top-down etching processes.^4^ A major drawback of many semiconductor lasers for applications in biological environments is their toxicity, especially when containing the element arsenic (As), which is predominant in many technologically relevant semiconductor alloys that emit in the range of 700–1500 nm.^14,15^ To ensure biocompatibility, these materials must be encapsulated, thereby requiring additional fabrication steps.^16^ The encapsulation layer also stabilizes the lasing wavelength, which is beneficial for cell tagging and tracking.^6^ On the other hand, it reduces the sensitivity to changes in the external refractive index, which is a challenge for sensing applications.

An alternative to As-based materials are alloys based on aluminum gallium indium phosphide (AlGaInP). These materials show much lower toxicity, offer attractive emission characteristics, and are compatible with all major nanofabrication procedures. Recently, we demonstrated that GaInP/AlGaInP quantum well structures are an excellent platform for biocompatible nanolasers.^4^ However, in our previous work, these lasers relied on optical pumping in the blue or green part of the visible spectrum due to their relatively high optical band gap of about 1.85 eV. These wavelength bands suffer from high absorption in most types of tissue (Figure 1a).^17^ Their use might thus cause photodamage to surrounding cells when used in thick samples. This is an important limitation as polymer-based biointegrated microlasers have recently been shown to outperform traditional microscopy-based methods in deep-tissue sensing of cardiomyocyte contractility.^8^

**Figure 1:**
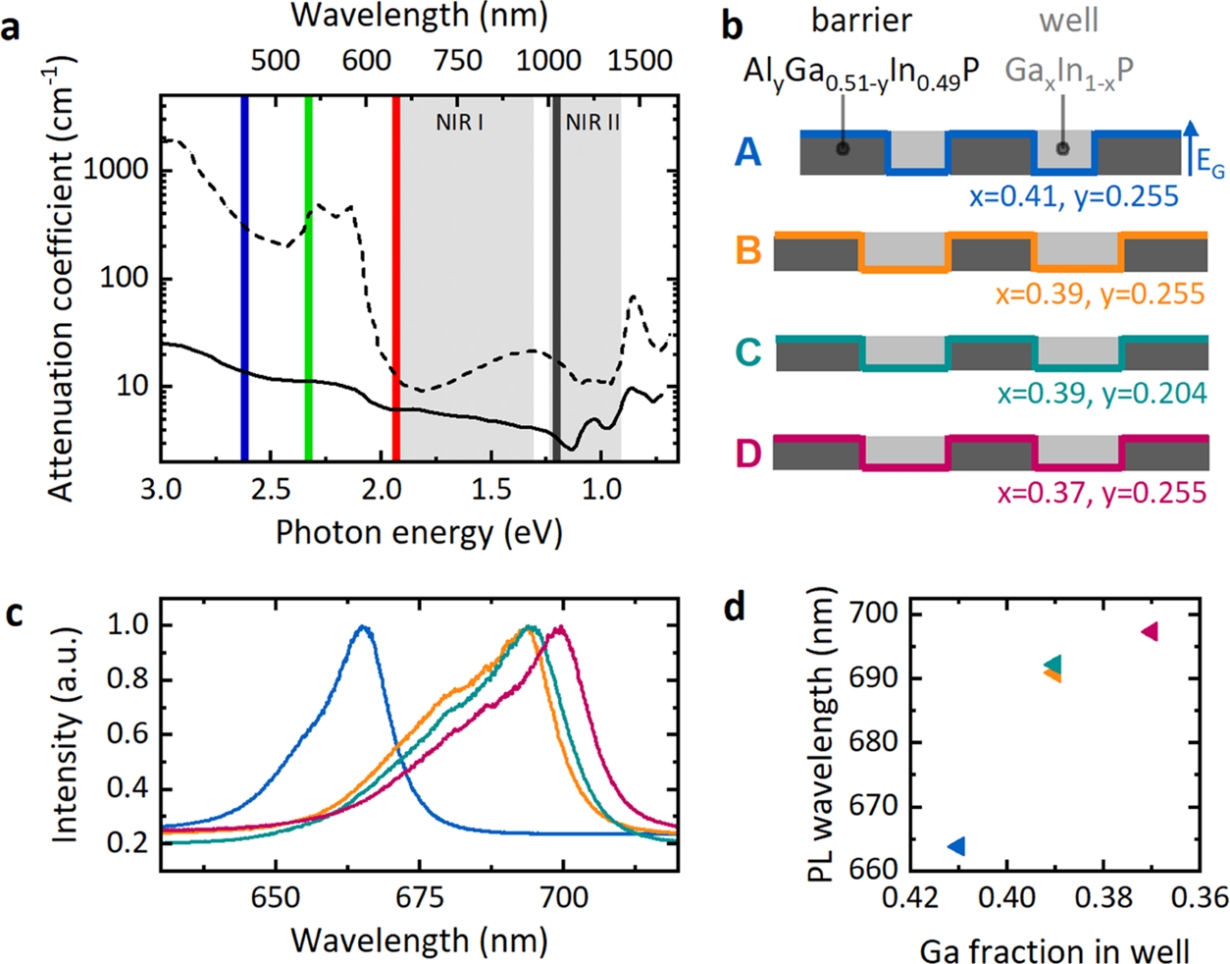
(a) Wavelength-dependent absorption in blood (dashed line) and skin (solid line), data from ref (17). The shaded regions show the NIR I and NIR II “therapeutic windows”, and the colored lines indicate different pump lasers that are compared in this work. (b) Schematic of the band gap and composition of GaInP quantum wells (light gray) with AlGaInP barriers (dark gray). The colored lines illustrate the evolution of the band gap *E*_G_ for each of the four tested wafers (A, B, C, and D). (c) Photoluminescence spectra of the four wafers, color-coded according to (b). (d) Peak photoluminescence wavelength versus gallium content of the active layer for each structure.

In this work, we address this issue by fine-tuning the AlGaInP multi-quantum well structure. Specifically, we adjusted the compositions and thicknesses of different layers of the multi-quantum well structure to engineer its band gap and thus the excitation and emission spectra. Microdisk lasers made from the optimized quantum well materials show low-threshold lasing across a broader range of pump wavelengths, extending into the “therapeutic windows”, which are wavelength bands with reduced scattering and absorption of light that are ideally suited for optical interrogation of biological tissue (Figure 1a).^17−19^ On the practical side, the broader excitation spectrum of new materials allows pumping of our microlasers with a commercially available pulsed diode laser that offers a higher repetition rate (up to 80 MHz) and lower cost than previously used solid-state pump lasers. In addition, we show that the microlasers developed here can be excited via two-photon excitation, which opens the possibility to interrogate biointegrated microlasers with multiphoton microscopes. Combining microdisk lasers with the extensive penetration depth of multiphoton microscopy holds great promise for novel deep-tissue cell tracking and sensing applications.

## Results and Discussion

The GaInP/AlGaInP quantum well structures were grown on GaAs substrates by metal–organic vapor-phase epitaxy (MOVPE). The multi-quantum well structure is sandwiched on each side by a 58 nm thick Al_0.375_Ga_0.153_In_0.49_P cladding and a 10 nm thick Ga_0.51_In_0.49_P buffer layer (Figure S1). The two active GaInP layers, which form the quantum wells, are interleaved with three AlGaInP barriers. The entire stack was grown onto a 700 nm thick Al_0.7_Ga_0.3_As sacrificial layer, which was removed during the microlaser fabrication process.

The thicknesses and compositions of barrier and well layers were systematically varied with the aim to reduce the optical band gap by shifting the energy levels of the system and thus allow pumping of our microlasers at longer wavelengths. The starting point for the optimization was the wafer structure used in our previous work,^4,10^ here referred to as Wafer A, consisting of 7 nm wide Ga_0.41_In_0.59_P wells and 10 nm Al_0.255_Ga_0.255_In_0.49_P barriers. Both an increase in well width and a decrease in the gallium content of the well are expected to lower the band gap and therefore to red-shift the photoluminescence.^20,21^ On the other hand, lower gallium concentrations lead to a greater mismatch in lattice constant compared to the GaAs substrate, and the resulting compressive strain increases the risk of forming dislocations in the structure.^22^ Furthermore, reducing the aluminum content in the barrier lowers its band gap,^23^ which enables absorption of longer wavelengths, however with the drawback of reduced electron confinement in the well.^24^ Due to these limitations, far-red emitting quantum-confined AlGaInP structures reported so far have suffered from significantly reduced performance.^25^

Weighing the different factors discussed above, three new multi-quantum well structures were fabricated: Wafer B (Ga_0.39_In_0.61_P wells, Al_0.255_Ga_0.255_In_0.49_P barriers), Wafer C (Ga_0.39_In_0.61_P wells, Al_0.204_Ga_0.306_In_0.49_P barriers), and Wafer D (Ga_0.37_In_0.63_P wells, Al_0.255_Ga_0.255_In_0.49_P barriers). In contrast to Wafer A, all new structures have 10 nm wide wells and barriers (Figure 1b). All structures in this study consist of two quantum wells, consistent with previously reported structures.^26^ We first measured the photoluminescence (PL) spectra of the four materials (Figure 1c). Fitting the center-most region of the photoluminescence spectrum of each wafer with a Gaussian revealed a peak emission wavelength of 664, 691, 692, and 697 nm for Wafers A, B, C, and D, respectively (Figure 1d). As expected, structures with less gallium and structures with wider quantum wells were found to emit further in the red. By contrast, the aluminum fraction in the barrier layer had little influence on the peak emission wavelength.^15,21^

**Figure 2:**
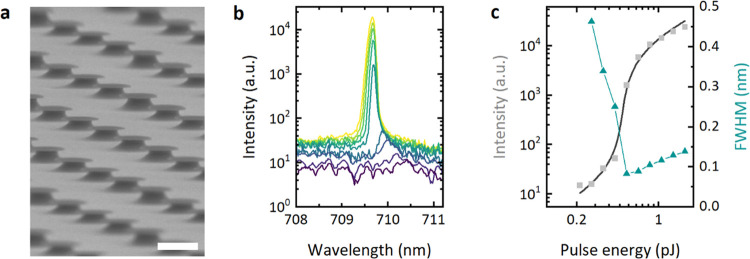
Laser fabrication and characterization. (a) Microdisk lasers supported on columns of the sacrificial layer after the HBr/Br_2_ etching step and prior to underetching by HF. Scale bar, 2 μm. (b) Emission spectra of a microdisk laser under 532 nm excitation. Pump pulse energy increases from purple to yellow. (c) Maximum output intensity (gray squares, left axis) versus pump pulse energy. The fit to the rate-equation model (black line) indicates a lasing threshold of 0.489 pJ. The linewidth of the lasing peaks (blue triangles, right axis) narrows near the lasing threshold and subsequently broadens at higher pump powers.

Disk-shaped whispering gallery mode microlasers with diameters ranging from 1.6 to 1.8 μm were fabricated from Wafers A, B, C, and D using UV lithography, reactive ion etching, and wet chemical etching (Figure 2a, see the Methods section). The nominal thickness of microdisks was 186 nm, except for disks made from Wafer A, which had a thickness of 180 nm. The weighted average refractive index of the multilayer structure was calculated to be 3.55.^27,28^

The lasing properties were investigated under pulsed excitation with a 532 nm solid-state laser (pulse duration, 727 ps; repetition rate, 125 kHz), which was expected to excite all structures reasonably efficiently. When increasing the energy of pump pulses, a narrow peak formed in the emission spectrum (Δλ_FWHM_ = 0.08 nm), which indicates the onset of lasing (Figure 2b). The microlasers studied predominantly showed single-mode emission with some rare cases of dual-mode emission. Profiles of the theoretical electric field distribution in a representative microlaser, along with its predicted emission spectrum, are shown in Figure S2. Analyzing the spectral width of the emission more closely revealed that the emission spectrum initially narrowed drastically before broadening again slightly with increasing pump pulse energy once the lasers were operated above the threshold (Figure 2c). In addition, we noticed an initial blue shift in the mode position coinciding with the linewidth narrowing (Figure S3), suggesting that carrier-dependent variations in modal gain and refractive index, which have been shown to cause broadening of the laser mode at higher pulse energies,^29,30^ are also responsible for this blue shift. The effect of carrier density on the modal gain of microlasers has been discussed in literature,^31,32^ where similar trends have been ascribed to the Burstein–Moss effect, where with increasing pump energy the apparent band gap blue-shifts due to band filling.^33^ We assume that, with increasing pump intensity, stimulated emission depletes the excited states more quickly, slowing down this effect and stabilizing the mode position well above the threshold.

To determine the lasing threshold of each laser, its input–output curve (Figure 2c) was fitted to the following rate equation for a semiconductor laser system^34^
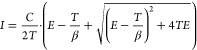
1with the spontaneous emission factor β, the cavity decay rate *T*, and an arbitrary constant *C* as free parameters, and *E* the pump pulse energy. The threshold pump pulse energy was calculated from the fitting parameters as
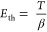
2

**Figure 3:**
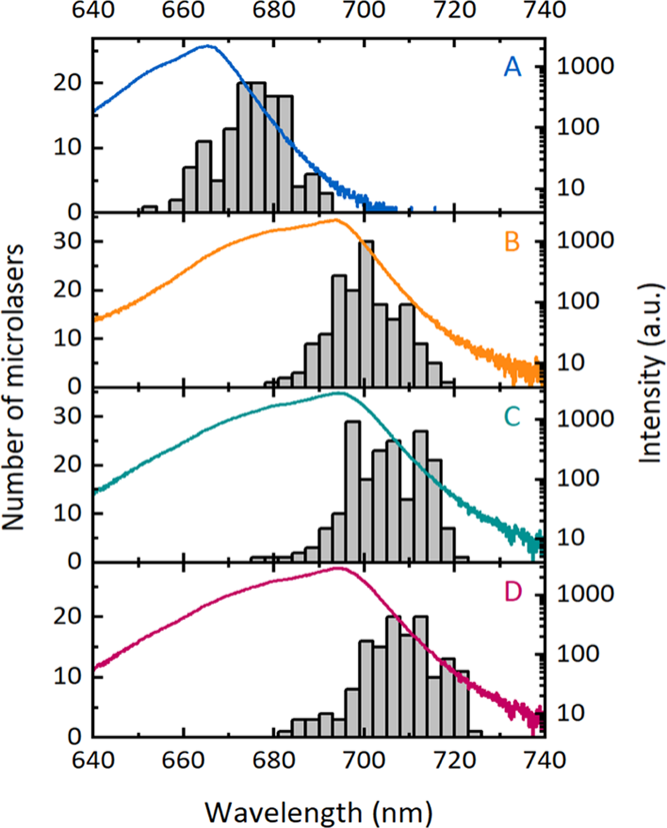
Bulk photoluminescence spectra for Wafers A, B, C, and D (in color, right axis) and histograms of lasing wavelength for microlasers fabricated from each wafer (left axis, *n* ≈ 150 for each wafer).

For pumping with 532 nm light, microlasers from all four compositions showed reliable lasing at low pump pulse energies. To establish the spectral range over which microdisk lasers made from the different materials operate, we acquired statistics on the distribution of lasing peaks for disks made from each of the four materials. Spectra recorded from approximately 150 microlasers were analyzed for each wafer by extracting the spectral position of the dominant mode using a Gaussian fit (Figure 3).

A strong correlation between the PL spectrum of different wafers and the spectral distribution of lasing peaks is found. Single-mode lasing generally appears at the long-wavelength shoulder of the PL spectrum, i.e., the peak of the histogram of lasing wavelength is red-shifted relative to the peak of the PL spectrum. This shift is least pronounced for Wafer B (9 nm), while Wafers A and D show a 12 nm shift and Wafer C has a 14 nm shift. The distribution of lasing peaks is a consequence of the mode competition at various microlaser diameters, which result from natural variations during the fabrication process. Therefore, the histograms of lasing wavelength visualize the effective optical gain distribution for each material, showing that the maximum gain is available at the long-wavelength shoulder of the PL spectrum where there is less reabsorption than at shorter wavelengths.

**Figure 4:**
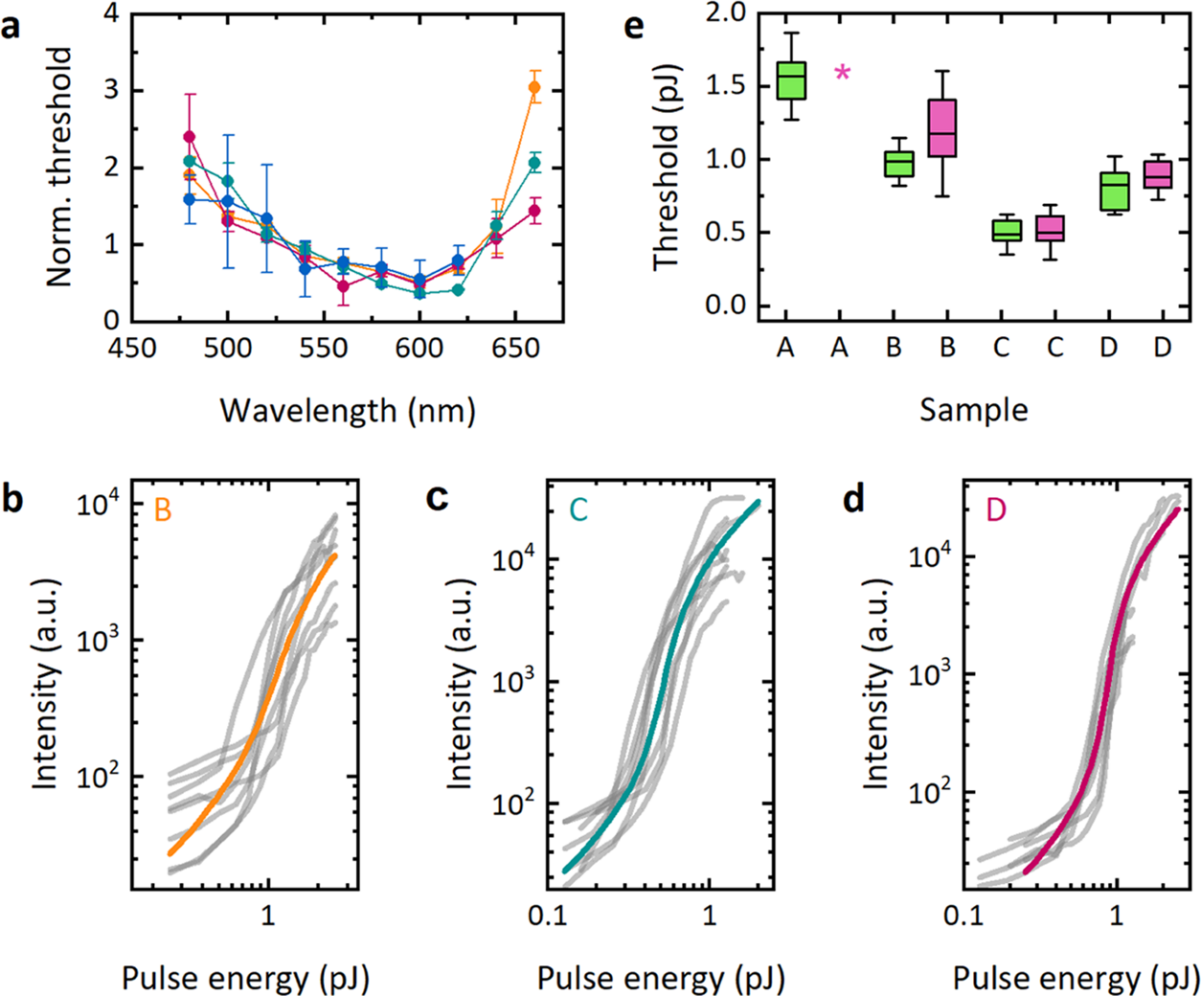
Dependence of lasing threshold on pump wavelength. (a) Normalized lasing thresholds of microlasers for different pump wavelengths. Error bars indicate the standard deviation. (b–d) Threshold characteristics at 642  nm, 10  MHz pumping for microdisk lasers made from Wafer B (b), Wafer C (c), and Wafer D (d). Thick lines in each panel represent threshold behavior predicted by the model for the average of the fitting parameters from each laser. (e) Statistics of a lasing threshold for pumping with 642 nm (pink) and 532 nm (green) light, showing mean threshold (center line), the 25–75 percentile (box), and 1.5 times the standard deviation (whiskers). Asterisk marks the sample where no lasing was detected.

To establish the spectral range in which different quantum well compositions can be excited, we investigated the dependence of the lasing threshold on the pump wavelength. Using an optical parametric oscillator (OPO), single microlasers were excited with light of different wavelengths between 480 and 660 nm in steps of 20 nm, and a threshold curve was obtained at each wavelength. The saturation regime of lasing was avoided to prevent any possible damage to the sample as this would affect the measurements at subsequent wavelengths. For each wafer, thresholds were recorded and averaged for at least 2 microlasers (Figure S4). The resulting threshold spectra (Figure S5) for each wafer were then normalized to the average threshold of the respective wafer in the spectral range between 480 and 620 nm, i.e., over the range of pump wavelengths that reliably yielded lasing for all wafers (Figure 4a). This normalization step allows to compare spectral trends despite the absolute thresholds being different between the four wafer compositions. Between 480 and 600 nm, the normalized threshold spectra are very similar, apart from Wafer C, which is expected to show improved absorption around 600 nm due to the reduced effective band gap of the barrier layer material. However, as the pump wavelength approaches the range of PL emission of each wafer, there is a steep increase in lasing threshold that is more rapid for the more blue-emitting structures, suggesting that in this spectral region, absorption stems from direct pumping of the quantum wells. Wafer A does not show consistent lasing when pumped at 640 and 660 nm, whereas all disks made from any of the three new wafers (B–D) show low-threshold lasing at 640 nm excitation. Above 640 nm, the shorter wavelength emitting Wafer B is found to have the steepest increase in the threshold between the three new materials, and Wafer D shows the lowest relative threshold. This shows that the absorption of the quantum wells has been shifted to the far-red spectral region, allowing to excite microdisk lasers efficiently at wavelengths that approach the NIR I therapeutic window.

We then measured threshold curves of multiple microdisk lasers from each wafer under excitation with the 532 nm solid-state laser and a compact 642 nm diode laser (operating at 10 MHz repetition rate, pulse duration 600 ps). Each threshold curve was fitted to the rate-equation model, and the fitting parameters for all individual threshold curves are listed in Tables S1 and S2. Figure 4b–d shows the experimental curves of Wafers B–D at 642 nm excitation, and the threshold behavior predicted by the model for the average of their fitting parameters. Microlasers from Wafer A did not lase when pumped with the 642 nm laser. The statistical variation in the threshold for microdisk lasers made from the different wafers is summarized in Figure 4e for both 642 and 532 nm pumping. The average lasing thresholds under 532 nm pumping were 1.60 ± 0.19, 0.99 ± 0.11, 0.49 ± 0.09, and 0.83 ± 0.13 pJ (*n* ≥ 6) for Wafers A–D, respectively. For 642 nm pumping, we obtained 1.18 ± 0.28, 0.51 ± 0.13, and 0.88 ± 0.10 pJ (*n* ≥ 7) for Wafers B–D, respectively (Figure 4e). As expected from the results obtained with the OPO pumping, all new wafer compositions could be efficiently pumped at 642 nm with similar thresholds as at 532 nm, whereas Wafer A did not lase under 642 nm excitation. Changing from green to red pumping, Wafer B showed an 18% increase in threshold, whereas no significant change was observed for Wafers C and D.

The two separate measurements with the tuneable OPO and with the 532 and 642 nm lasers confirm that the newly designed wafers are excitable at longer wavelengths. Comparing the wafers for which only the quantum well composition was varied, lower gallium content clearly red-shifts the absorption along with the emission. Interestingly, Wafer C shows a very similar PL spectrum compared to Wafer B, but more readily absorbs longer wavelengths. We attribute the improved absorption of Wafer C to higher light absorption in the barrier due to the decreased aluminum content. This red-shift in absorption also explains the larger difference between PL and lasing maxima of Wafer C (Figure 3) since reabsorption might prevent optical gain across a larger portion of the PL spectrum.

**Figure 5:**
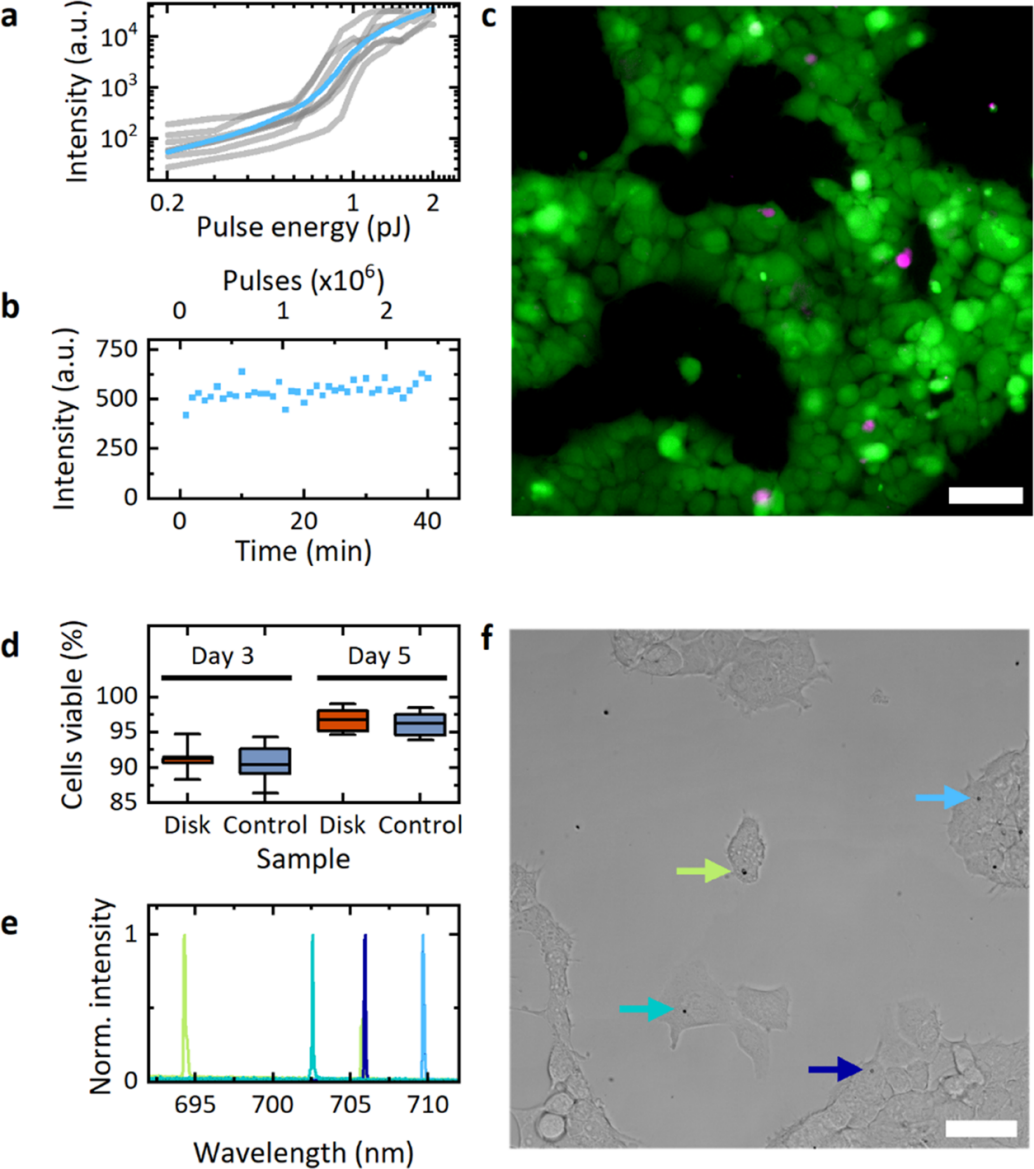
(a) Threshold curves and (b) stability of laser emission under continuous pumping in an aqueous environment. (c) Epifluorescence microscopy image of HEK293 cells incubated with microdisk lasers for 3 days. Staining with cell tracker dye (green, live cells) and propidium iodide (magenta, dead cells). Scale bar, 40 μm. (d) Statistical analysis of cell staining showing no decrease in the viability of cells incubated with microlasers for 3 and 5 days compared to control cultures without microlasers. Mean (center line), 25–75 percentile (box) and 1.5 times the standard deviation (whiskers). (e) Normalized lasing spectra of intracellular lasers after 3 days of incubation. (f) Brightfield microscope image of HEK293 cells incubated with microdisk lasers. Arrows point to intracellular microlasers with color-coding corresponding to spectra in (e). Scale bar, 50 μm.

Next, microlasers were tested for their stability and biocompatibility. We first investigated the performance in aqueous environments. Lasing thresholds of microdisk lasers made from Wafer D were measured under 642 nm pumping at 10 MHz repetition rate, while the lasers rested on the bottom of a dish filled with deionized water (Figure 5a). The lasing threshold calculated from fitted curves (Table S3) was 0.90 ± 0.12 pJ (*n* = 7), very similar to the 0.88 ± 0.10 pJ measured in air. We conclude that due to the high refractive index of the disk lasers, their mode confinement is not significantly affected by the index difference between air and water. The stability in deionized water was investigated while pumping at 1 kHz and well above the threshold (Figure 5b). After 40 min of continuous pumping, corresponding to 2.4 million pulses, no visible degradation was observed. Here, the small fluctuations in the amplitude of laser emission (Δ*I*_RMS_ = 8.02%) were likely due to the instability of the pump laser (Δ*E*_RMS_ = 9.30%). We further note that in this configuration, the orientation of microdisk lasers was such that signal collection occurred along the direction of least efficient emission;^35^ therefore, we expect that any rotations of the microlasers during cell tracking experiments will only increase the lasing signal.

In the next experiment, the biocompatibility of the lasers was investigated using a cell culture model. A live–dead assay was conducted after incubating HEK293 cells with microdisk lasers for 3 and 5 days, respectively (Figure 5c). There was no detectable decrease in viability of cells with microlasers compared to control samples on either day (Figure 5d).

We also collected intracellular lasing spectra (Figure 5e), as well as corresponding brightfield microscopy images of microdisk-containing cells (Figure 5f, day 3). In addition to indicating that the direct contact of cells with the microdisks has negligible effects on their viability, this experiment also demonstrated that our microlasers can operate for multiple days in cell culture medium and when inside living cells.

**Figure 6:**
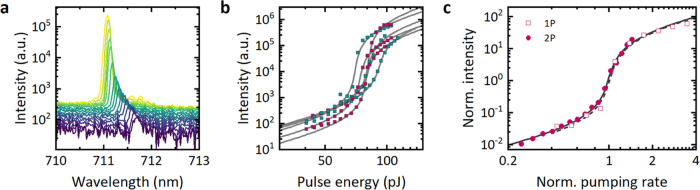
(a) Lasing spectra under two-photon excitation with power increasing from purple to yellow. (b) Input–output characteristics for several microdisks prepared from Wafer C (cyan) and Wafer D (magenta) and corresponding fits to the modified rate-equation model. (c) Rescaled threshold curves under 532 nm single-photon (squares, dashed line) and 1030 nm two-photon excitation (dots, solid line) from Wafer D microlasers. The pump rate was calculated from the linear (single-photon) and power-squared (two-photon) dependence of the excited fluorescence, respectively. The curves were normalized such that at the threshold, pump rate and output power are 1.

Microlasers from the two lowest threshold materials, Wafer C and Wafer D, were further used to study lasing behavior under two-photon excitation. A 1030 nm source (pulse duration, 250 fs; repetition rate, 10 kHz) was used for pumping. As under single-photon excitation, the lasing mode first blue-shifted and then stabilized with increasing power (Figure 6a), and the width of the emission spectrum was narrowest (Δλ_FWHM_ = 0.07 nm) around the lasing threshold (Figure S6a).

The quadratic intensity dependence of two-photon absorption resulted in a much steeper input–output curve than for single-photon pumping (Figures 6b and S6b). To describe the two-photon excitation process more accurately, we modified our original rate equation to include a pumping rate proportional to the square of pump pulse energy (Note S1). Fitting the input–output data with this modified rate equation resulted in β factors of order 10^–3^, similar to the single-photon case. Hence, after rescaling the pumping rate to account for the linear and quadratic dependencies of absorption under single- and two-photon pumping, respectively, the threshold curves had almost identical shapes (Figure 6c). This indicates that the decay and lasing dynamics that follow the excitation are independent of the excitation process itself, thus likely allowing to predict two-photon thresholds from those measured under sub-nanosecond single-photon pumping.

The absolute lasing thresholds for two-photon pumping were 81 ± 11 pJ for Wafer C and 78 ± 4 pJ for Wafer D (determined from the fitting parameters, *n* = 3, Table S4). Thus, compared to single-photon pumping, the thresholds for two-photon pumping were about 2 orders of magnitude higher (Figure S6c), although we note that the differences between the two pump configurations, in terms of pump pulse duration and shape of the pump spot, prevented a detailed quantitative comparison of the differences in the threshold. An increased lasing threshold under two-photon pumping is expected due to the generally reduced absorption cross-section. In fact, compared to conventional fluorescence microscopy, two-photon microscopy also generally requires higher excitation powers combined with the use of ultrashort pulses to generate sufficient signals.^36,37^ Literature on two-photon pumping of lasers is limited, but the existing examples, e.g., CdSe/CdS nanorod lasers,^38^ ZnO nanowires,^39^ and CsPbBr_3_ spheres,^40^ also show substantially increased thresholds under two-photon pumping. However, it is important to note that the lasing thresholds observed in our experiments are 2–3 orders of magnitude lower than the pulse energies available from the ultrafast laser systems used in state-of-the-art commercial multiphoton microscopes (typically 3 W average power, 80 MHz repetition rate) and well within the range of pulse energies proven to be safe for repeated two-photon microscopy studies of biological specimens (approx. 10 mW^41−43^ at 80 MHz repetition rate, which corresponds to 125 pJ/pulse).

## Conclusions

In summary, we presented a comparative study of microlasers from four different GaInP/AlGaInP multi-quantum well structures. Increasing the width of the quantum wells and lowering their gallium content reduced the band gap and therefore red-shifted the emission and absorption of microdisk lasers fabricated from these modified materials. The lower band gaps enabled direct sub-picojoule optical pumping with a pulsed diode laser operating at 642 nm for all new materials presented. Reducing the aluminum content in the barrier further improved absorption at longer wavelengths. Since the optical gain in the materials is affected by reabsorption, changes in the barrier composition also shifted the distribution of lasing peaks further away from the PL emission. The improved performance of the optimized quantum well structures implies that emission and absorption could be shifted even further to the red by increasing the strain beyond what has been explored in the current study, at least for microscopic devices for which structural defects and dislocations can be tolerated to a certain extent.

Furthermore, the GaInP/AlGaInP multi-quantum well-material system minimizes toxicity as confirmed via tests in a simple cell culture model. In addition, we did not observe degradation of microdisk lasers after prolonged exposure to physiological conditions, i.e., in cell culture medium and when internalized by cells. We thus expect that they are compatible with long-term studies without a need for additional encapsulation, which maximizes refractive index sensitivity as required for biosensing applications.

Finally, we found that our stable, low-threshold microlasers can be excited via two-photon absorption, and we expect that, with further optimization, the two-photon excitation of semiconductor microdisk lasers can be rendered more efficient. Even at the present stage, the lasing thresholds of our microdisk lasers are well within the reach of commercial two-photon microscopes and within the photon budget of in vivo microscopy in terms of photodamage and phototoxicity. By allowing efficient pumping in the red or near-infrared spectral region, both the excitation and the emission of microlasers now sit in an optical transparency window favorable for biointegration and are compatible with direct pumping from electrically driven diode lasers, rather than requiring more expensive, bulky, and less robust solid-state lasers. This will simplify the integration of microlaser-based cell tagging and sensing approaches into conventional confocal and two-photon microscopes. In conclusion, the improved material design and compatibility with two-photon pumping greatly enhance the scope and flexibility of biointegrated microlasers, including for future use in deep-tissue sensing and multiphoton microscopy.

## Methods

### Microlaser Fabrication

The wafers were grown on GaAs substrates by metal–organic vapor-phase epitaxy (MOVPE) at the EPSRC National Epitaxy Facility in Sheffield, U.K. Substrates were cleaned by 3 min sonication in IPA, acetone, deionized water, and methanol, followed by 3 min of O_2_ plasma. We then spin-coated a ∼380 nm thick layer of SU8 photoresist (SU8 2000.5, KayakuAM, 3:1 dilution with cyclopentanone), which was soft baked on a hot plate at 90 °C for 2 min. A pattern of filled circles was exposed with UV lithography, using a mask with 3 μm diameter holes, followed by a 2 min post exposure bake at 90 °C. The resist was developed in 2-methoxy-1-methylethyl acetate (EC solvent, Microposit) for 60 s and then cured at 180 °C for 10 min. A 30 s descumming step with reactive ion etching in oxygen plasma was performed, followed by a 12 s wet etch in the Br_2_/HBr solution. The SU8 caps were then removed by reactive ion etching in oxygen plasma for 7 min. The remaining disk structures rested on columns of the sacrificial layer, which were selectively etched away in 5% hydrofluoric acid for 3 min. During this step, the detached microlasers collapsed onto the GaAs substrate from where they were readily harvested into a Petri dish upon sonication in an aqueous environment, leaving free-floating microlasers in suspension.

### Optical Characterization

Microlaser performance was characterized using one of two custom-built micro-PL setups constructed on inverted microscopes. For the threshold comparison with green and red excitation, 532 nm (Coherent Helios 532-4-125) and 642 nm (Omicron Quixx 642-140 PS) pump lasers were coupled into the microscope to form a diffraction limited excitation spot. The microlaser signal was collected using a 25× silicone immersion objective (Nikon CFI Plan Apochromat Lambda S 25XC Sil). On the second setup, pump lasers operating at 473 nm (Alphalas Pulselas-P-473-10, used for cell experiments) and 1030 nm (Toptica FemtoFiber Vario 1030) and a tuneable OPO (Opotek Opolette) were used for microlaser excitation. A back focal plane lens was added to form a collimated excitation beam (*d* ≈ 200 μm). In this second setup, either a 60× oil immersion objective (Nikon Plan Apo VC) or a 40× air objective (Nikon S Plan Fluor ELWD) was used. Both microscopes were connected to a spectrograph (Andor Shamrock 500i) equipped with an 1800 lines/mm grating and an EM-CCD camera (Andor Newton). The optical resolution of the spectrometer was approximately 40 pm.

### Live–Dead Assay

Four dishes with HEK293 cells were cultured in medium (Dulbecco’s modified Eagle’s medium, fetal bovine serum, penicillin/streptomycin, glutamine) and incubated at 37 °C. For microlaser samples, a piece of a wafer with fully underetched microlasers was sonicated in an Eppendorf tube in phosphate-buffered saline (PBS) to detach microlasers. The microlaser suspension was diluted with cell medium and added to the respective dish through a filter with a pore size of 5 μm. The dishes were then incubated for either 3 or 5 days, with medium replaced after the third day in the two dishes designated for the 5 day stability test. For the fluorescent imaging, cells were stained with green cell tracker dye (5-chlormethylfluorescein diacetate, Fisher Scientific Cat# C2925, 10 μM) for 30 min, with DAPI (4′,6-diamidino-2-phenylindole, dilactate, Fisher Scientific Cat# 11530306, 25 μg/mL) for 5 min, and with propidium iodide (Calbiochem, Cat# 537059, 1.5 μM) for 5 min and then imaged immediately. Epifluorescence images for the live–dead assay were obtained on an inverted epifluorescence microscope (Nikon Eclipse Ti) using a 40× or 20× objective (Nikon S Plan Fluor ELWD). Cells were counted manually with the ImageJ cell tracker plugin, using the DAPI stain to count the total number of cells (day 3, *n* > 1400; day 5, *n* > 3400) and discriminating between live (cell tracker) and dead (propidium iodide) cells from the epifluorescence images.

## Supporting Information Available

Epitaxial design of wafers; simulations of microlaser modes; additional measurements of laser linewidth, emission wavelength, and wavelength-dependent threshold pump fluence; rate-equation model and threshold pulse energy measurements of two-photon lasing; and tables with fitting parameters for single- and two-photon rate-equation models (PDF)

## Notes

The research data supporting this publication can be accessed at https://doi.org/10.17630/f7fde0bc-7ca7-4634-a58e-a0844243e1d6.
