# Supplementary material for: Red-Shifted Excitation and Two-Photon Pumping of Biointegrated GaInP/AlGaInP Quantum Well Microlasers
Source: ACS Photonics. 2022 Feb 16;9(3):952–60. doi: 10.1021/acsphotonics.1c01807 (PMC9007562; doi:10.1021/acsphotonics.1c01807)
Supplement: Supplementary file 1 — ph1c01807_si_001.pdf [file ph1c01807_si_001.pdf]

Supporting Information

Red-shifted excitation and two-photon pumping of biointegrated GaInP/AlGaInP quantum well microlasers

Vera M. Titze[a], Soraya Caixeiro[a], Andrea Di Falco[a],

Marcel Schubert[a,b*], and Malte C. Gather[a,b*]

[a] SUPA, School of Physics and Astronomy, University of St Andrews, North Haugh, St Andrews, KY16 9SS, United Kingdom

[b] Humboldt Centre for Nano- and Biophotonics, Institute of Physical Chemistry, University of Cologne, Greinstr. 4-6, D-50939 Cologne, Germany

*E-mail: malte.gather@uni-koeln.de, marcel.schubert@uni-koeln.de

This document (page S1-S10) includes:

- Figures S1 to S6
- Tables S1 to S4
- Note S1

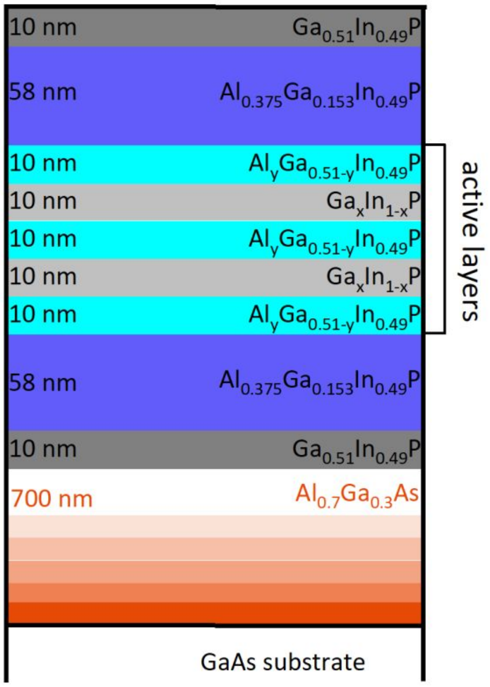

Figure S1 Composition and thickness of the wafer structure used in this study. On top of the GaAs substrate (white), a 700 nm-thick $Al_{0.7}Ga_{0.3}As$ sacrificial layer (shaded orange) is grown first, followed by the different layers that form the microlaser structure. The active layers of the multi-quantum well structure are sandwiched on both sides by a 58 nm-thick $Al_{0.375}Ga_{0.153}In_{0.49}P$ cladding (purple) and a 10 nm-thick $Ga_{0.51}In_{0.49}P$ buffer layer (dark grey). The compositions of the GaInP wells (light grey) and the AlGaInP barriers (cyan) are varied between Wafers A, B, C, and D. The thicknesses shown correspond to Wafers B-D, while the wells of Wafer A are only 7 nm thick.

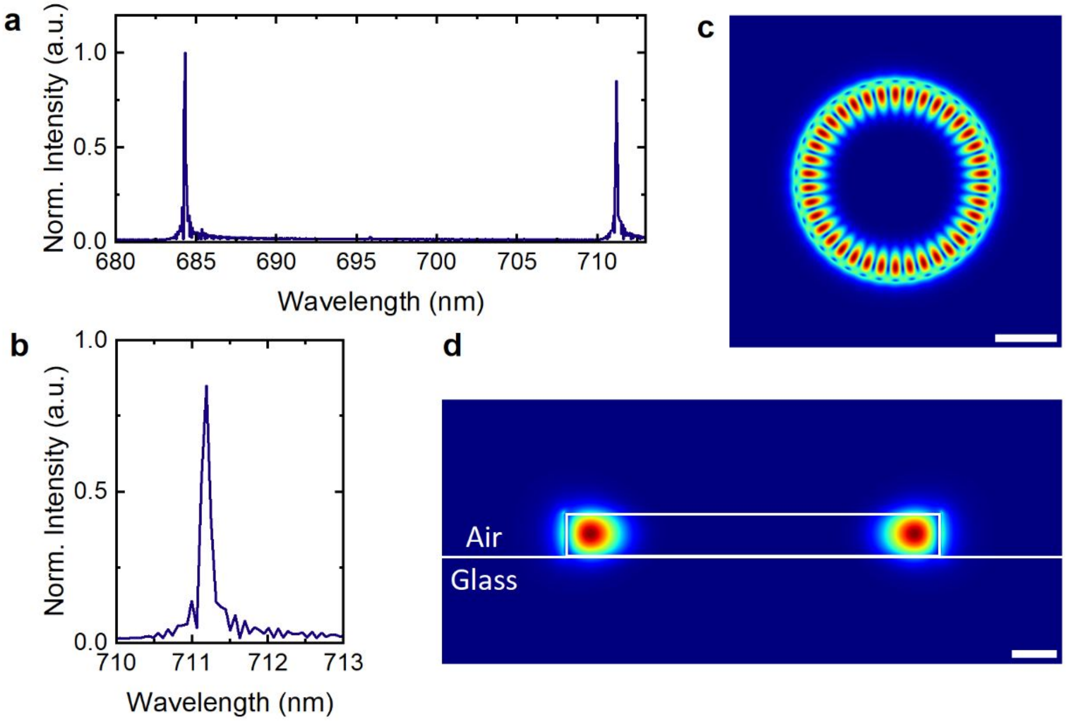

Figure S2 Numerical modelling of microlasers. a) Modelled emission spectrum of 1.68 µm diameter microlaser in air, resting on glass, with peaks corresponding to modes TE_{19} (711 nm) and TE_{20} (684 nm). b) Close-up of TE_{19} peak, showing a linewidth of 0.13 nm. c) Electric field distribution of the TE_{19} mode in the plane of the microdisk. Scale bar, 500 nm. d) Electric field distribution of the TE_{19} mode over the cross-section through the disk, showing the geometry of the microlaser (white box) on the glass substrate. Scale bar, 200 nm. Modelling was performed with 3D finite-difference time-domain (FDTD) simulation (Ansys Lumerical), using a 2.6 µm (x, y) by 1 µm (z) mesh with a spatial resolution of 0.006 µm, and perfectly matched layer (PML) boundary conditions. The simulation time was 15000 fs with a timestep of 0.0104 fs.

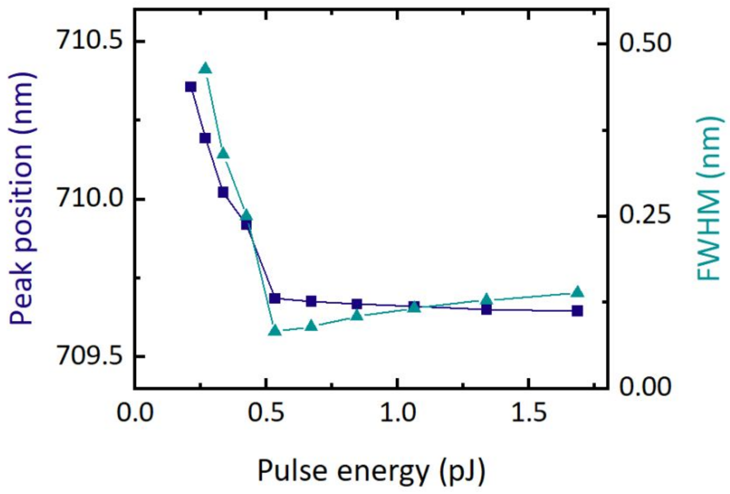

Figure S3 Peak position (blue squares, left axis) and full width at half maximum (FWHM, cyan triangles, right axis) of the lasing spectrum as function of pump pulse energy for the threshold curve shown in Fig. 2. Below threshold, the peak position shows a blue shift and concomitant linewidth narrowing. Above threshold (0.489 pJ), the peak position becomes stable.

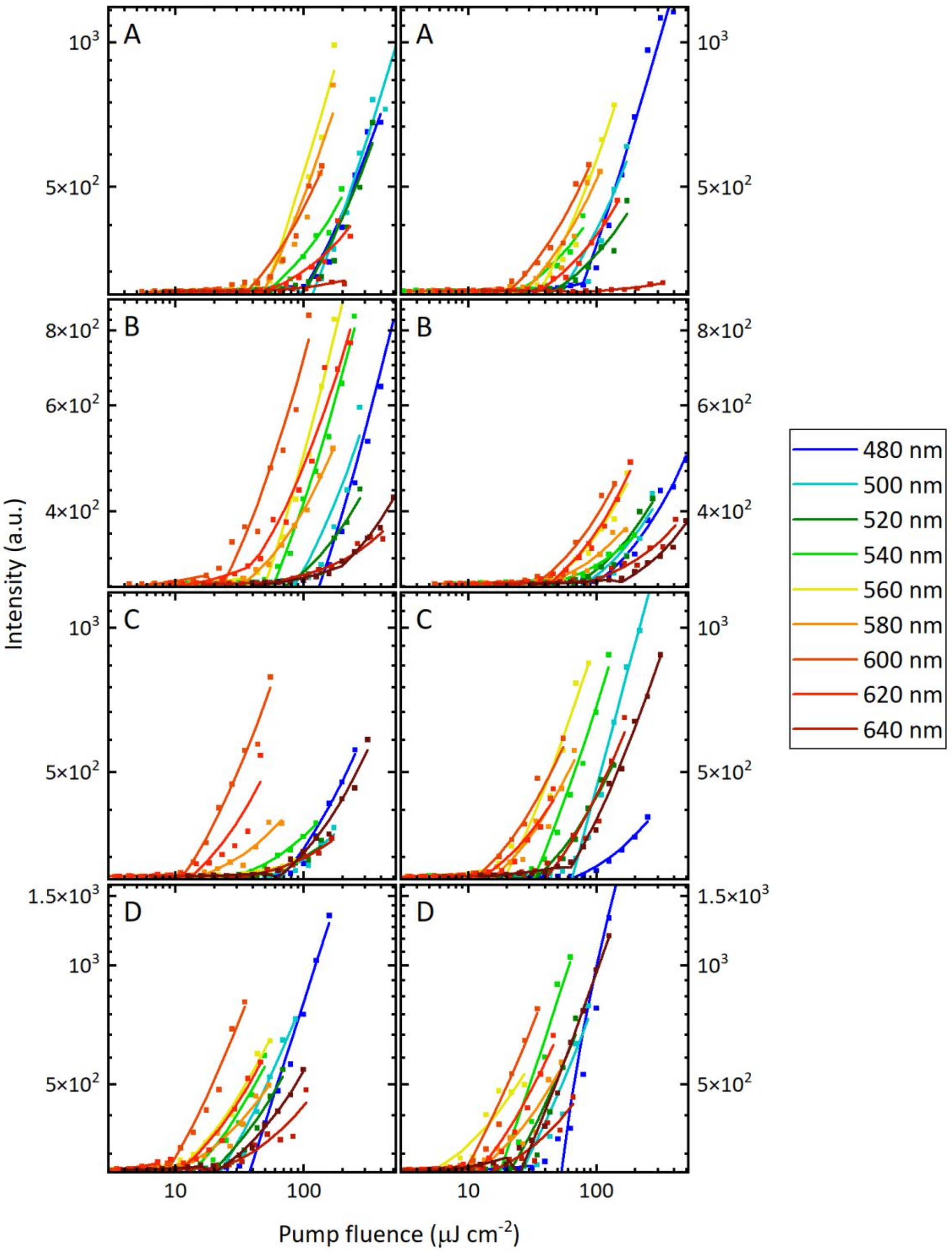

Figure S4 Wavelength-dependent lasing thresholds for each wafer composition. Shown are the input-output curves for two disks from each wafer, color-coded for 10 different pump wavelengths from 480 nm (blue) to 660 nm (maroon). A double linear fit (solid lines in corresponding colors) was applied to each curve to calculate the lasing threshold. This method was applied because the microlasers were not excited far enough above threshold to allow reliable fitting with the rate equation model.

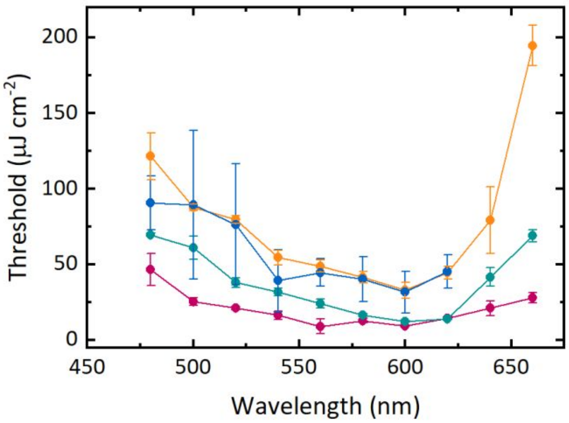

Figure S5 Wavelength-dependent lasing thresholds calculated from data shown in Fig. S4 prior to normalization (see Fig. 4a in main text for normalized data).

Note S1 Rate Equation for Two-Photon Excitation

The two-photon rate equation was derived from the rate equation[1] for the single-photon absorption

$$y = \frac{C}{2T} \cdot \left(x - \frac{T}{\beta} + \sqrt{\left(x - \frac{T}{\beta} \right)^2 + 4Tx} \right),$$

where the pumping rate x was assumed to be proportional to the pulse energy of the pump. In the case of two-photon excitation, the following substitution was applied to reflect the power-squared dependence of two-photon excited fluorescence:

$$x = aE^2,$$

where a represents the relative absorption cross section for two-photon absorption. Consequently, the lasing threshold is calculated from the fitting parameters as

$$E_{th} = \sqrt{\frac{T}{a\beta}}.$$

As shown in Figure S3, the adjusted rate equation model shows excellent agreement with the experimental input-output curve. Moreover, the β factors obtained for single- and two-photon pumping are comparable when applying the appropriate model for each case (Tables S1 and S4). This suggest that despite the different pumping processes, the emission dynamics are similar in both cases.

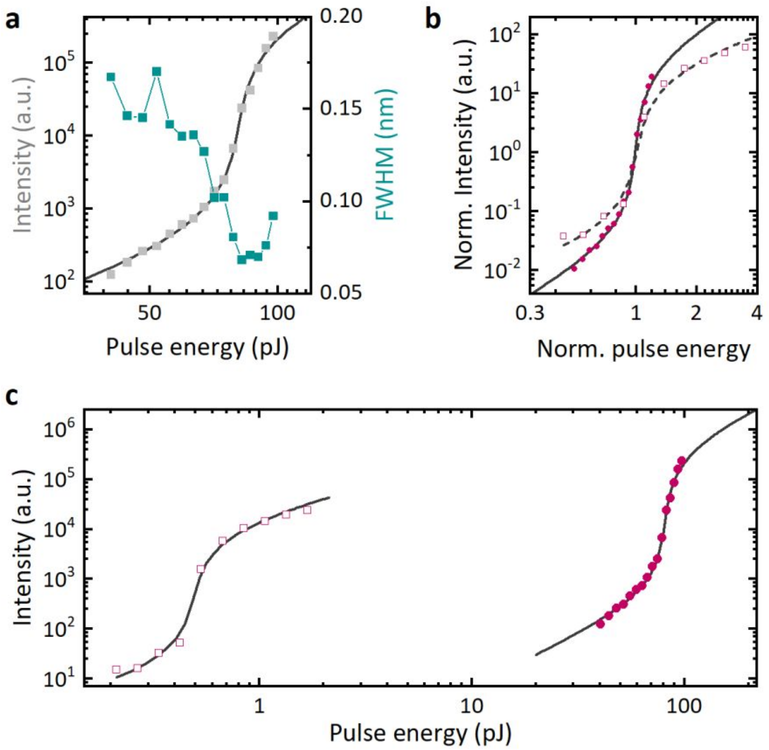

Figure S6 a) Input-output curve (gray squares, left axis) for the spectra shown in Fig. 6a of the main text and corresponding calculated full width half maximum of peaks (cyan squares, right axis). b) Normalized threshold curves for single- (open symbols) and two-photon (closed symbols) excitation plotted over a normalized pump energy to show the steeper increase in output power of the two-photon-pumped microlaser. Solid lines represent fits to the rate equation model assuming a linear (single-photon) and a power-square (two-photon) dependence of excitation rate on the pulse energy of the pump laser, respectively. c) Threshold curves from (b) on an absolute scale.

Table S1 Fitting Parameters under 532 nm excitation

	FWHM (nm)	T	C	β	E_{th} (pJ)
A-1	0.15	0.0094	146	0.0070	1.34
A-2	0.11	0.0178	166	0.0092	1.94
A-3	0.13	0.0028	42	0.0017	1.64
A-4	0.13	0.0033	39	0.0020	1.69
A-5	0.11	0.0036	25	0.0024	1.51
A-6	0.13	0.0041	48	0.0027	1.49
A-7	0.17	0.0065	51	0.0040	1.61
B-1	0.09	0.0049	22	0.0054	0.89
B-2	0.08	0.0021	38	0.0019	1.08
B-3	0.10	0.0013	16	0.0014	0.87
B-4	0.14	0.0022	13	0.0022	1.03
B-5	0.12	0.0055	29	0.0053	1.03
B-6	0.10	0.0033	25	0.0028	1.16
B-7	0.14	0.0049	17	0.0046	1.05
B-8	0.14	0.0024	27	0.0028	0.86
B-9	0.17	0.0030	29	0.0033	0.90
C-1	0.08	0.0008	8	0.0019	0.45
C-2	0.08	0.0004	7	0.0011	0.36
C-3	0.10	0.0010	28	0.0017	0.60
C-4	0.11	0.0022	54	0.0038	0.58
C-5	0.08	0.0005	14	0.0011	0.49
C-6	0.08	0.0009	25	0.0019	0.46
D-1	0.13	0.0043	40	0.0048	0.91
D-2	0.10	0.0011	12	0.0016	0.97
D-3	0.11	0.0020	38	0.0022	0.91
D-4	0.09	0.0016	20	0.0021	0.80
D-5	0.11	0.0020	13	0.0030	0.66
D-6	0.09	0.0009	9	0.0014	0.64
D-7	0.11	0.0010	18	0.0011	0.91

Table S2 Fitting parameters under 642 nm excitation

	T	C	β	E_{th} (pJ)
B-1	0.0180	181	0.0179	1.62
B-2	0.0270	160	0.0270	1.10
B-3	0.0351	111	0.0322	1.09
B-4	0.0076	36	0.0077	0.99
B-5	0.0491	62	0.0462	1.06
B-6	0.0279	102	0.0378	0.74
B-7	0.0241	57	0.0181	1.33
B-8	0.0562	78	0.0377	1.50
C-1	0.0041	126	0.0062	0.66
C-2	0.0028	71	0.0068	0.42
C-3	0.0046	126	0.0095	0.48
C-4	0.0032	49	0.0067	0.48
C-5	0.0029	136	0.0060	0.49
C-6	0.0072	92	0.0124	0.58
C-7	0.0074	72	0.0112	0.66
C-8	0.0022	21	0.0078	0.28
D-1	0.0042	33	0.0058	0.72
D-2	0.0034	44	0.0038	0.89
D-3	0.0030	20	0.0034	0.89
D-4	0.0030	77	0.0029	1.03
D-5	0.0031	48	0.0037	0.85
D-6	0.0031	77	0.0038	0.81
D-7	0.0031	55	0.0031	0.99

Table S3 Fitting parameters under 642 nm excitation, measured in de-ionised water

	T	C	β	E_{th} (pJ)
D-1	0.0118	312	0.0154	0.77
D-2	0.0030	246	0.0037	0.80
D-3	0.0020	198	0.0037	1.02
D-4	0.0096	207	0.0019	0.99
D-5	0.0036	72	0.0097	1.04
D-6	0.0027	83	0.0035	0.80
D-7	0.0080	177	0.0085	0.94

Table S4 Fitting parameters under 1030 nm excitation

	FWHM (nm)	α	T	C	β	E_{th} (pJ)
C-1	0.07	0.00025	0.0019	467	0.0012	80.8
C-2	0.19	0.00009	0.0002	242	0.0004	70.0
C-3	0.15	0.00040	0.0063	690	0.0019	91.7
D-1	0.14	0.0003	0.0007	434	0.00033	81.2
D-2	0.15	0.0628	0.2711	150	0.00079	73.8
D-3	0.17	0.0014	0.0053	105	0.00062	78.9

References

(1) Rice, P. R.; Carmichael, H. J. Photon Statistics of a Cavity-QED Laser: A Comment on the Laser Phase-Transition Analogy. *Phys. Rev. A* **1994**, *50* (5), 4318–4329
